# *Orientia tsutsugamushi* modulates cellular levels of NF-κB inhibitor p105

**DOI:** 10.1371/journal.pntd.0009339

**Published:** 2021-04-15

**Authors:** Tanaporn Wangsanut, Katelynn R. Brann, Haley E. Adcox, Jason A. Carlyon

**Affiliations:** Department of Microbiology and Immunology, Virginia Commonwealth University Medical Center, School of Medicine, Richmond, Virginia, Unites States of America; LSU School of Veterinary Medicine, UNITED STATES

## Abstract

**Background:**

Scrub typhus is a neglected tropical disease that threatens more than one billion people. If antibiotic therapy is delayed, often due to mis- or late diagnosis, the case fatality rate can increase considerably. Scrub typhus is caused by the obligate intracellular bacterium, *Orientia tsutsugamushi*, which invades phagocytes and endothelial cells *in vivo* and diverse tissue culture cell types *in vitro*. The ability of *O*. *tsutsugamushi* to replicate in the cytoplasm indicates that it has evolved to counter eukaryotic host cell immune defense mechanisms. The transcription factor, NF-κB, is a tightly regulated initiator of proinflammatory and antimicrobial responses. Typically, the inhibitory proteins p105 and IκBα sequester the NF-κB p50:p65 heterodimer in the cytoplasm. Canonical activation of NF-κB via TNFα involves IKKβ-mediated serine phosphorylation of IκBα and p105, which leads to their degradation and enables NF-κB nuclear translocation. A portion of p105 is also processed into p50. *O*. *tsutsugamushi* impairs NF-κB translocation into the nucleus, but how it does so is incompletely defined.

**Principal findings:**

Western blot, densitometry, and quantitative RT-PCR analyses of *O*. *tsutsugamushi* infected host cells were used to determine if the pathogen’s ability to inhibit NF-κB is linked to modulation of p105. Results demonstrate that p105 levels are elevated several-fold in *O*. *tsutsugamushi* infected HeLa and RF/6A cells with only a nominal increase in p50. The *O*. *tsutsugamushi*-stimulated increase in p105 is bacterial dose- and protein synthesis-dependent, but does not occur at the level of host cell transcription. While TNFα-induced phosphorylation of p105 serine 932 proceeds unhindered in infected cells, p105 levels remain elevated and NF-κB p65 is retained in the cytoplasm.

**Conclusions:**

*O*. *tsutsugamushi* specifically stabilizes p105 to inhibit the canonical NF-κB pathway, which advances understanding of how it counters host immunity to establish infection.

## Author summary

Scrub typhus is a neglected disease that can be fatal and occurs predominantly in the Asia-Pacific, one of the most densely populated regions of the world. Notably, cases continue to emerge outside this area. The etiologic agent is *Orientia tsutsugamushi*, a bacterial pathogen that infects certain leukocytes and cells that line blood vessels in animals and humans. The success of *O*. *tsutsugamushi* to colonize these cells is at least partially attributable to its ability to counter host immunity. In this study, we demonstrate that *O*. *tsutsugamushi* stabilizes p105, a mammalian inhibitor of the transcription factor, NF-κB, which is otherwise key for activating proinflammatory and antimicrobial gene expression. *O*. *tsutsugamushi* is the first example of a bacterium that inhibits NF-κB by promoting elevated levels of p105 and impairing its degradation. Our findings provide fundamental information that helps explain how this important pathogen has evolved to stealthily establish infection in host cells.

## Introduction

Scrub typhus is a serious but neglected zoonosis long known to be endemic to a geographic area referred to as the tsutsugamushi triangle, which encompasses Asia, northern Australia, and islands of the western Pacific and Indian oceans (reviewed in [1,2]). The World Health Organization designated scrub typhus one of the world’s most underdiagnosed/underreported diseases that often requires hospitalization [1]. The etiologic agent is *Orientia tsutsugamushi*, an obligate intracellular bacterium that is vectored by *Leptotrombidium* spp. mites. More than one billion people are at risk for infection within the tsutsugamushi triangle and roughly one million new cases are estimated to occur annually [1,2]. Reports of scrub typhus cases, seroprevalence of antibodies against *O*. *tsutsugamushi* antigens, and detection of *O*. *tsutsugamushi* DNA in rodents and *Leptotrombidium* spp. mites indicate the presence of the pathogen in African countries and Chile [2–10]. A new species, *O*. *chuto*, was recently recognized as the etiologic agent of a scrub typhus-like illness in the United Arab Emirates [11]. These *Orientia* species constitute an expanding global health threat. When transmitted to its natural mammalian reservoirs or accidental human hosts, *O*. *tsutsugamushi* invades phagocytes and endothelial cells [2]. Consequently, scrub typhus pathogenesis typically presents in highly vascularized organs and can manifest as fever, rash, vasculitis, pneumonitis, myopericarditis, as well as liver and kidney disease [1,2]. If left untreated, disease can progress to systemic vascular collapse and multi-organ failure with fatality rates that can reach as high as 70% [2]. The ability of *O*. *tsutsugamushi* to replicate to high numbers in the cytoplasm suggests that it has evolved to counter immune defense mechanisms as part of its strategy for surviving within diverse eukaryotic hosts.

NF-κB is an evolutionarily conserved immune defense molecule, the activation of which is the central initiating cellular event of host responses to microbes. The pleiotropic transcription factor upregulates expression of more than 500 genes involved in the antimicrobial response, inflammation, and cell function (reviewed in [12–14]). Mice lacking NF-κB are highly susceptible to bacterial, viral, and parasitic infections [13]. The NF-κB family consists of hetero- or homodimeric combinations of five members: RelA (p65), RelB, c-Rel, NF-κB1 (p105/p50), and NF-κB2 (p100/p52). NF-κB dimers are retained in the cytoplasm by forming complexes with members of a family of inhibitory proteins known as inhibitors of NF-κB (IκBs). The IκB family consists of IκBα, IκBβ, IκBδ, p100, and p105. The best characterized NF-κB dimer is p50:p65, which is activated through the canonical (classical) pathway. In resting cells, p50:p65 is sequestered in the cytoplasm as a latent complex by its physical association with IκBα. TNFα, IL-1, or bacterial products activate inhibitory κB kinase β (IKKβ) to phosphorylate key serines of IκBα, triggering its polyubiquitination and degradation by the 26S proteasome. The ensuing release of NF-κB enables it to translocate into the nucleus and activate gene expression [12,13].

In addition to IκBα, p105 sequesters p50:p65 in the cytoplasm. p105 is also a precursor of p50. The *NFκB1* gene encodes both p105 and p50. p105 is a 971-amino acid protein of which residues 1–430 constitute p50 [12]. p50 generation from p105 occurs by both co- and post-translational mechanisms. Co-translation of p50 and p105 from *NFκB1* is mediated by the 26S proteasome to yield both proteins from a single mRNA [15]. p50 post-translational generation occurs by constitutive 20S proteasomal processing of p105 in a ubiquitin-independent manner [15,16]. Like IκBα, when the classical NF-κB pathway is activated, p105 is serine-phosphorylated by IKKβ, polyubiquitinated, and degraded by the 26S proteasome to release NF-κB for nuclear translocation [17–20]. Mice deficient in p105 develop spontaneous lymphocytic inflammation in the lung and liver [21], indicating its importance as a suppressor of inflammation.

*O*. *tsutsugamushi* impairs the classical NF-κB response by inhibiting nuclear accumulation of p50:p65 in both resting and TNFα-stimulated cells [22], the responsible mechanisms of which need to be further defined. Moreover, although IκBα degradation proceeds unhindered in *O*. *tsutsugamushi* infected cells [22], the status of p105 is unknown. This study examined p105 stability in *O*. *tsutsugamushi* infected cells under resting conditions and activation of the classical NF-κB pathway. Results demonstrate that the bacterium post-transcriptionally increases p105 cellular levels with minimal effect on p50. Even though TNFα-induces phosphorylation of p105 serine 932 (Ser932) in infected cells, p105 levels remain elevated and NF-κB p50:p65 is retained in the cytoplasm. This work advances understanding of how *O*. *tsutsugamushi* impairs a critical component of the antimicrobial response.

## Methods

### Cell lines and cultivation of *O*. *tsutsugamushi*

HeLa human cervical epithelial cells (CCL-2; American Type Culture Collection [ATCC], Manassas) were maintained in Roswell Park Memorial Institute (RPMI) 1640 medium supplemented with 10% (vol/vol) fetal bovine serum (FBS; Gemini Bio-Products, Sacramento, CA, USA) at 37°C in a humidified incubator with 5% CO_2_ prior to *O*. *tsutsugamushi* infection. RF/6A rhesus monkey choroidal endothelial cells (ATCC CRL-1780) were cultivated in Dulbecco’s Modified Eagle’s Medium (DMEM; Invitrogen, Carlsbad, CA) supplemented with 10% FBS, 2 mM L-glutamine, 1x MEM Non-Essential Amino Acids (Invitrogen), and 15 mM HEPES. *O*. *tsutsugamushi* str. Ikeda, which was originally isolated from a patient in Japan [23], was maintained and propagated every 3 to 4 days in HeLa cells in RPMI 1640 medium supplemented with 1% FBS and 1X Anti-Anti (Thermo Fisher Scientific, Waltham, MA) at 35°C in a humidified incubator with 5% CO_2_. To obtain *O*. *tsutsugamushi* for infection studies, highly infected HeLa cells were mechanically lysed using glass beads followed by centrifugation at 200 x *g* for 5 min to remove cellular debris. The supernatant was centrifuged at 2,739 x *g* for 10 min, which yielded a bacterial cell pellet that was resuspended in fresh medium for experimental use. To initiate synchronous infection in HeLa or RF/6A cells, medium was removed at 2 to 4 h after inoculation and replaced with fresh medium. In infection experiments, naïve cells were infected with *O*. *tsutsugamushi* at a multiplicity of infection (MOI) of 10 unless otherwise stated. MOI was confirmed by fixing an aliquot of infected cells per experiment and visualizing using immunofluorescence microscopy as previously described [22]. In some experiments, uninfected or infected *O*. *tsutsugamushi* samples were treated with 10 μg ml^-1^ of oxytetracycline hydrochloride (Sigma-Aldrich, St. Louis, MO) or vehicle control (70% ethanol) at 4, 24, and 48 h post-infection followed by collection of the cells at 72 h. In some cases, uninfected and infected cells were treated with TNFα (10 ng/mL) (Life Technologies, Grand Island, NY) or vehicle control (0.1% bovine serum albumin [BSA] in H_2_O) at 72 h post-infection for 30 or 60 min (to assess p105-induced degradation) or 5 min (to assess and IKKβ-induced Ser932 phosphorylation). For experiments designed to examine p105-induced phosphorylation, calyculin A (50 nM) (Cell Signaling Technology, Danvers, MA) was added simultaneously with TNFα.

### Western blotting, antibodies, and reagents

Cells were washed with 1X PBS, scraped, centrifuged at 10,000 x *g* for 10 min, and cell pellets were lysed in radioimmunoprecipitation assay buffer (RIPA) containing Halt Protease Phosphatase Inhibitor Cocktail (Thermo Fisher Scientific). After a 45- to 120-min incubation on ice, clarified lysates were obtained by centrifugation at 16,000 x *g* for 10 min followed by retention of the supernatant. Protein Assay Reagent (Bio-Rad, Hercules, CA) was used to quantify protein concentration. Whole cell lysates and cellular fractions (5–15 μg per lane) were resolved by SDS-PAGE in 4 to 20% Mini-Protean gels (Bio-Rad) as described previously [24]. Blots were probed with antibodies in tris-buffered saline with Tween-20 (TBS-T: 25 mM Tris HCl, 137 mM NaCl, 2.7 mM KCl, 0.05% Tween-20; pH 7.4) containing 5% non-fat milk or 5% BSA. Western blot analyses were performed using the following primary antibodies: mouse anti-glyceraldehyde-3-phosphate dehydrogenase (GAPDH) (catalog number sc-365062, Santa Cruz, Dallas, TX) at a 1:750 dilution; rabbit anti-IκBα (9242, Cell Signaling Technology, Danvers, MA) at a 1:1,000 dilution; rabbit anti-p105 (ab131546, Abcam, Cambridge, UK) at a 1:750 dilution; rabbit anti-p105/p50 (ab32360, Abcam) at a 1:1,000 dilution; rabbit anti-phospho-p105 (4806, Cell Signaling Technology) at a 1:1,000 dilution; mouse anti-p65 (sc-8008, Santa Cruz) at a 1:250 dilution; rabbit anti-*O*. *tsutsugamushi* TSA56 (56-kDa type-specific antigen) [25] at a 1:1,000 dilution; rabbit anti-Lamin A/C (2032, Cell Signaling Technology) at a 1:1,000 dilution; rabbit anti-IKKα antibody (2682, Cell Signaling Technology); rabbit anti-IKKβ antibody (2370, Cell Signaling Technology); and rabbit anti-TNFR1 (21574-1-AP, Proteintech) at a 1:500 dilution. Secondary antibodies used to detect bound primary antibodies were horseradish-peroxidase-conjugated anti-mouse or anti-rabbit IgG (7076 or 7074, respectively; Cell Signaling Technology) at 1:10,000 dilution. All blots were incubated with SuperSignal West Pico, SuperSignal West Dura, or SuperSignal West Femto chemiluminescent substrate (Thermo Fisher Scientific), and visualized using the ChemiDoc Touch Imaging System (Bio-Rad). Densitometry analysis was determined from at least three separate blots using Bio-Rad Image Lab software.

#### Immunofluorescence microscopy

HeLa cells were seeded onto glass coverslips within 24-well plates and infected with *O*. *tsutsugamushi* at a MOI of 10, 20, or 50. At 48 h, cells were fixed and permeabilized with -20°C methanol. All wash steps were performed using PBS. Coverslips were blocked in 5% (vol/vol) bovine serum albumin (BSA) in PBS for 1 h at room temperature prior to incubation with rabbit antiserum against TSA56. Coverslips were washed three times before incubation with Alexa Fluor 488-conjugated goat anti-rabbit IgG at a 1:1,000 dilution in 5% BSA for 1 h at room temperature. Samples were incubated with 0.1 μg ml^-1^ 4’ 6-diamidino-2-phenylindole (DAPI, Invitrogen) in PBS for 1 min, washed three times, and mounted using ProLong Gold Antifade mounting media (Invitrogen). Coverslips were imaged with a Zeiss LSM 700 spinning disc confocal microscope using a 63X oil immersion objective (Zeiss, Oberkochen, Germany). Images were acquired using ZEN Black software (Zeiss) and ImageJ macro Fiji [26, 27] was used to analyze the captured images. The percentage of infected cells was determined by counting 100 cells per coverslip and determining the mean + SD of infected cells for triplicate samples.

### Cytosolic and nuclear fractionation

HeLa cells (70% confluency) were incubated with *O*. *tsutsugamushi* at a MOI of 10 or with uninfected HeLa cells as the mock infection control. At 72 h, spent media was removed and replaced with fresh media containing TNFα (10 ng/mL) or vehicle control for 30 min or 60 min. Media was removed and the cells were rinsed with 1X PBS, dislodged by scraping, and pelleted by centrifugation at 200 x *g* for 4 min. The cells were rinsed and subjected to a second round of centrifugation. Cytosolic and nuclear fractions were obtained using the Nuclear Extraction Kit (Abcam) and analyzed by Western blot.

### RNA isolation and quantitative real-time PCR

HeLa cells were mock infected or infected with *O*. *tsutsugamushi* at a MOI of 10. Samples were collected at 24, 48, and 72 h by scraping and centrifugation as described above. Total RNA was extracted using the RNeasy minikit (Qiagen, Germantown, MD). Amplification-grade DNase (Invitrogen) was added to RNA samples to remove genomic DNA. The iScript Supermix cDNA synthesis kit (Bio-Rad) was used to convert 1 μg of each RNA sample into cDNA. PCR using human GAPDH gene-specific primers (5’-ACATCATCCCTGCCTCTACTGG-3’ and 5’-TCCGACGCCTGCTTCACC-3’) was performed to confirm DNA-free RNA samples. qRT-PCR was performed using SsoFast EvaGreen supermix (Bio-Rad). Primers targeting *NFκB1* were 5’-TTCTGGACCGCTTGGGTAAC-3’ and 5’-CGTTGGGGTGGTCAAGAAGT-3’. Primers specific for the *O*. *tsutsugamushi* 16S rRNA gene were 5’-GTGGAGCATGCGGTTTAATTCGATGATC-3’ and 5’- TAAGAATAAGGGTTGCGCTCGTTGC-3’. Relative gene expression levels of *NFκB1* and the *O*. *tsutsugamushi* 16S rRNA gene were normalized to human *GAPDH* transcript levels using the 2^-ΔΔ*CT*^ method [28].

### Statistical analysis

The Student’s t-test was performed to test for a significant difference among pairs using Prism (version 7.0) software package (GraphPad, San Diego, CA). Statistical significance was set to *P* < 0.05.

## Results

### p105 levels are elevated in *O*. *tsutsugamushi* infected cells under resting state conditions

**Fig 1:**
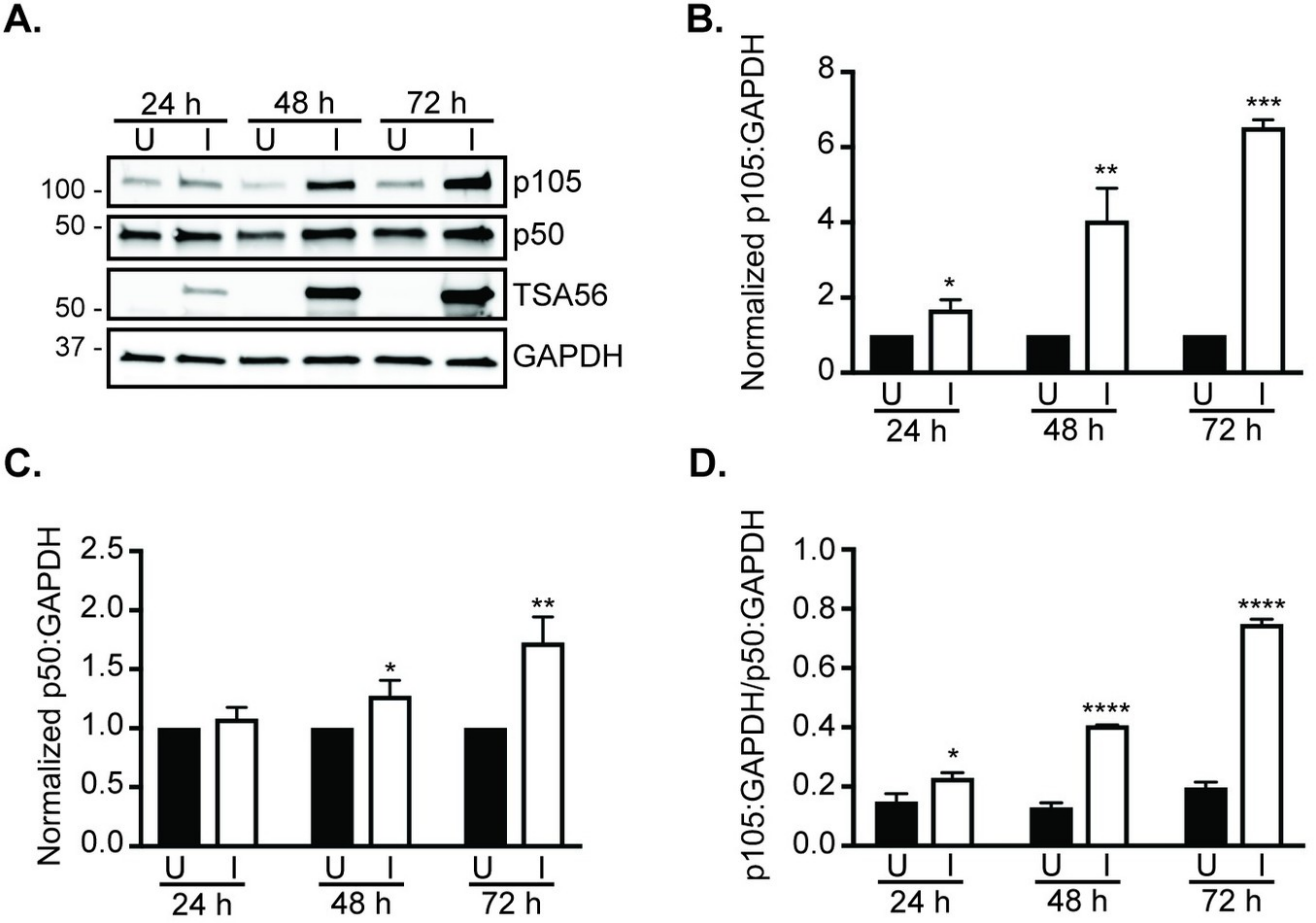
p105 levels are increased in *O*. *tsutsugamushi* infected HeLa cells under resting state conditions. (A) Whole cell lysates of *O*. *tsutsugamushi* infected (I) and mock infected (U) HeLa cells recovered at 24, 48, and 72 h post-infection were subjected to Western blot analyses using antibodies specific for p105, p50, *O*. *tsutsugamushi* TSA56, and GAPDH. Data are representative of three separate experiments. (B to D) Mean normalized ratios + SD of p105:GAPDH (B), p50:GAPDH (C), and p105:GAPDH/p50:GAPDH (D) at the indicated time point(s) from three independent experiments were calculated using densitometry. Statistically significant (*, *P* < 0.05; **, *P* < 0.01; ***, *P* < 0.001; ****, *P* < 0.0001) values are indicated.

**Fig 2:**
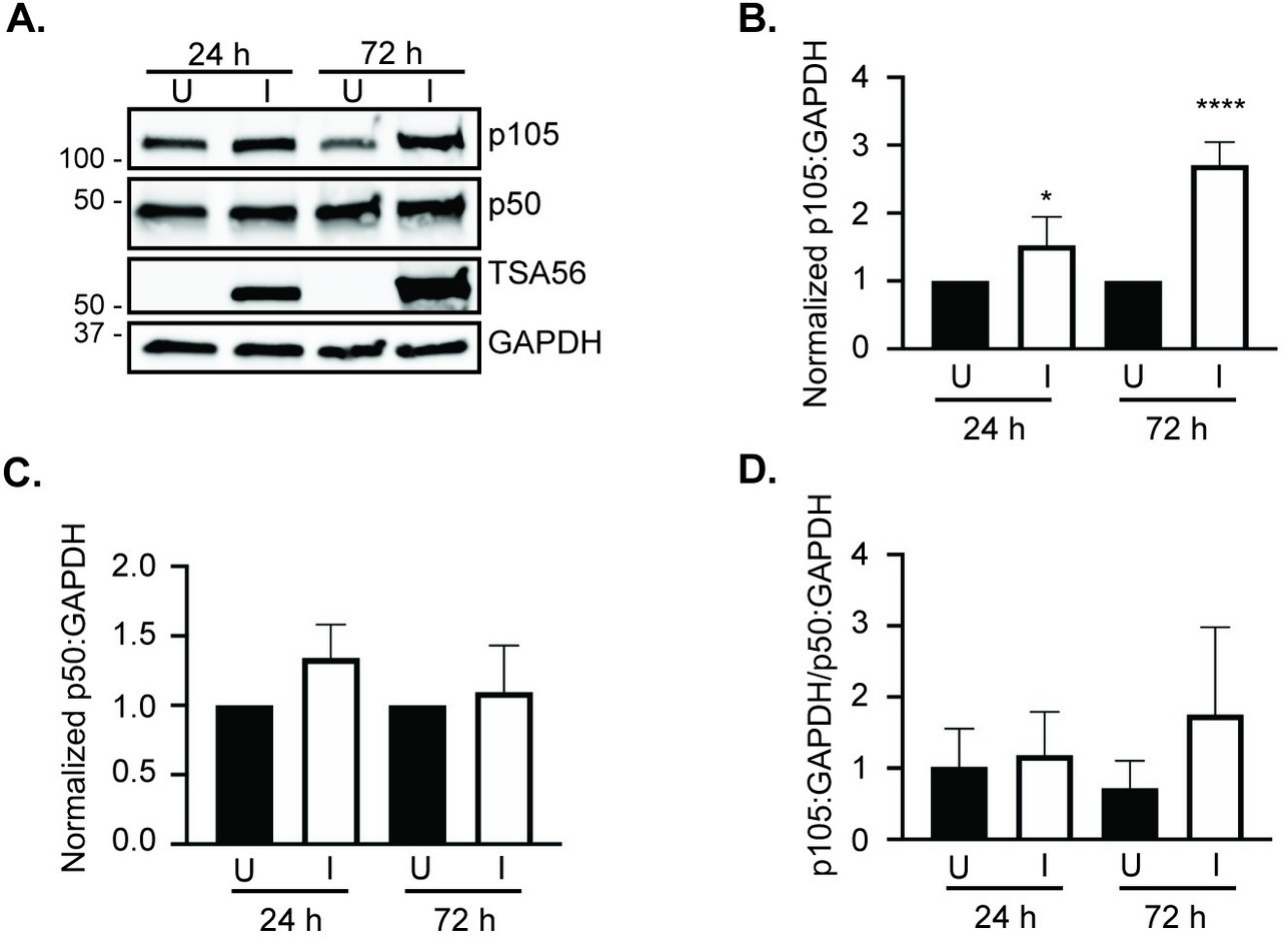
p105 levels are elevated in *O*. *tsutsugamushi* infected RF/6A endothelial cells under resting state conditions. (A) Whole cell lysates of *O*. *tsutsugamushi* infected (I) and mock infected (U) RF/6A cells recovered at 24 and 72 h post-infection were subjected to Western blot analyses using p105, p50, TSA56, and GAPDH antibodies. Data are representative of three separate experiments. (B to D) Mean normalized ratios + SD of p105:GAPDH (B), p50:GAPDH (C), and p105:GAPDH/p50:GAPDH (D) from three independent experiments was calculated using densitometry. Statistically significant (*, *P* < 0.05; ****, *P* < 0.0001) values are indicated.

To determine if p105 levels are altered over the course of *O*. *tsutsugamushi* infection under resting state conditions, HeLa cells, which are routinely used to study *O*. *tsutsugamushi*-host cell interactions [22,25,29–36], were infected at a MOI of 10. Whole cell lysates recovered at 24, 48, and 72 h were examined for p105 and p50 via Western blot and densitometric analyses. Probing with antibody against the bacterium’s immunodominant outer membrane protein, TSA56 [25,37], confirmed that the appropriate samples were infected and that the pathogen load increased over the time course (Fig 1A). GAPDH antibody was used to verify that equal protein amounts had been loaded per condition. p105 levels were significantly elevated in infected cells compared to uninfected cells at all time points and by more than six-fold at 72 h (Fig 1A and 1B). Increases in p50 levels at 48 and 72 h were not nearly as pronounced (Fig 1A and 1C). Dividing the levels of p105 normalized to GAPDH by p50 levels normalized to GAPDH revealed that *O*. *tsutsugamushi* infection results in an approximate three-to-one ratio of p105 to p50 (Fig 1D). Because *O*. *tsutsugamushi* exhibits a tropism for endothelial cells, the experiment was repeated using primate-derived RF/6A endothelial cells. Comparable results to those for HeLa cells were observed (Fig 2). Thus, *O*. *tsutsugamushi* infection leads to a robust increase in p105 cellular levels.

### The *O*. *tsutsugamushi*-induced increase in p105 is bacterial dose-dependent, bacterial protein synthesis-dependent, but is not due to increased *NFκB1* transcription

**Fig 3:**
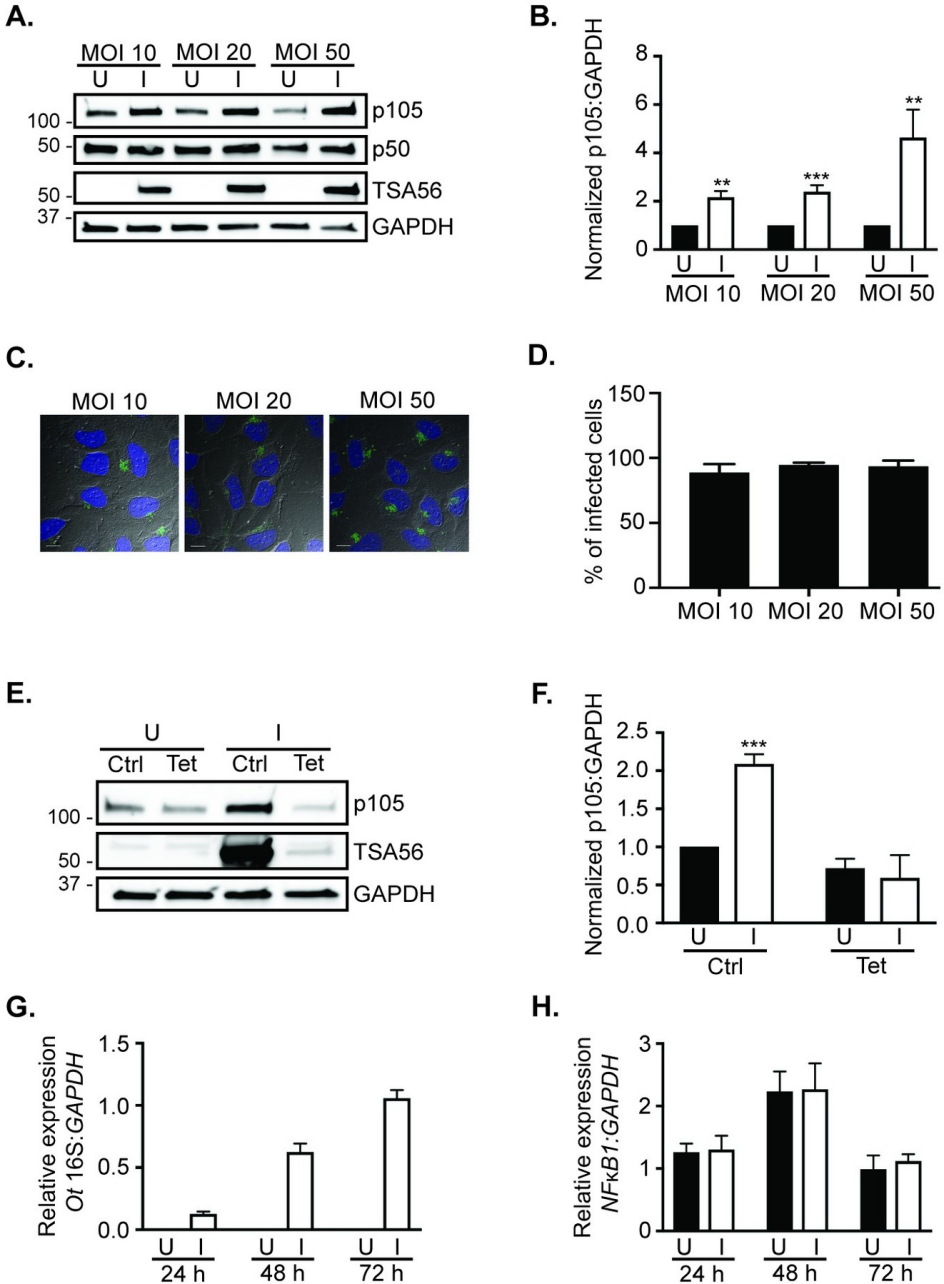
*O*. *tsutsugamushi* increases p105 levels in bacterial dose- and protein synthesis-dependent manners, but does not increase *NFκB1* transcription. (A to D) HeLa cells were infected with *O*. *tsutsugamushi* (I) at a MOI of 10, 20, or 50 for 48 h. Mock infected HeLa cells (U) were a negative control. Whole cell lysates were subjected to Western blot analyses using the indicated antibodies (A). The mean normalized ratios + SD of p105:GAPDH from three independent experiments in (A) were calculated using densitometry (B). Data are representative of three separate experiments. Also at 48 h, cells were fixed and permeabilized with methanol, screened with TSA56 antiserum, stained with DAPI, and visualized using spinning-disk confocal microscopy. (C) Presented are merged fluorescence images overlaid on differential interference contrast images of the same fields of view. Scale bars, 10 μm. (D) The mean + SD percentage of infected cells were determined by counting 100 cells for five separate coverslips per condition. (E and F) Whole cell lysates of U or I (MOI of 10) HeLa cells that had been incubated in the presence of oxytetracycline (Tet) or vehicle control (Ctrl) or mock infected controls (U) were subjected to Western blot (E) analyses using the indicated antibodies at 72 h post-infection. Data are representative of three separate experiments. The mean normalized ratios + SD of p105:GAPDH from three independent experiments in (F) were calculated using densitometry. (G and H) Total RNA isolated at 24, 48, or 72 h from triplicate samples of U or I HeLa cells were subjected to qRT-PCR analyses. The 2^-ΔΔ*CT*^ method was used to determine the relative *O*. *tsutsugamushi* 16S rRNA gene (*Ot* 16S) (G) or *NFκB1* (H) expression normalized to that of *GAPDH*. Data are indicative of similar results from three separate experiments each performed in triplicate. Statistically significant (**, *P* < 0.01; ***, *P* < 0.001) values are indicated.

To determine if bacterial load influences the increase in p105, HeLa cells were incubated with *O*. *tsutsugamushi* at MOIs of 10, 20, and 50. At 48 h, p105 levels were increased by four-fold in cells that had been infected with a MOI of 50 versus two-fold rises in cells infected with 10 or 20 organisms per cell (Fig 3A and 3B). Immunofluorescence microscopy examination of culture aliquots at 48 h confirmed that equivalent percentages of the cells were infected regardless of the initial MOI (Fig 3C and 3D). To assess whether the pathogen’s ability to modulate p105 levels is protein synthesis-dependent, tetracycline or vehicle control was added to *O*. *tsutsugamushi* infected HeLa cells beginning at 4 h. When examined at 72 h, significant elevation of p105 was no longer observed for cells that had been treated with tetracycline (Fig 3E and 3F). Next, it was evaluated whether *NFκB1* gene expression is higher during *O*. *tsutsugamushi* infection. Total RNA isolated from infected and uninfected HeLa cells was analyzed by quantitative reverse transcription-PCR using primers targeting *NFκB1*, *O*. *tsutsugamushi* 16S rRNA, and *GAPDH*. *O*. *tsutsugamushi* 16S rRNA levels increased only in infected samples over the time course, thereby confirming that the infection proceeded normally (Fig 3G). No increase in *NFκB1* mRNA levels in infected cells compared to uninfected controls was observed at any time point of infection (Fig 3H). Together, these data establish that *O*. *tsutsugamushi* increases p105 levels in bacterial load- and tetracycline-sensitive manners without eliciting a concomitant increase in *NFκB1* transcription.

### *O*. *tsutsugamushi* prevents TNFα-induced degradation of p105, but not IκBα to retain NF-κB in the host cell cytoplasm

**Fig 4:**
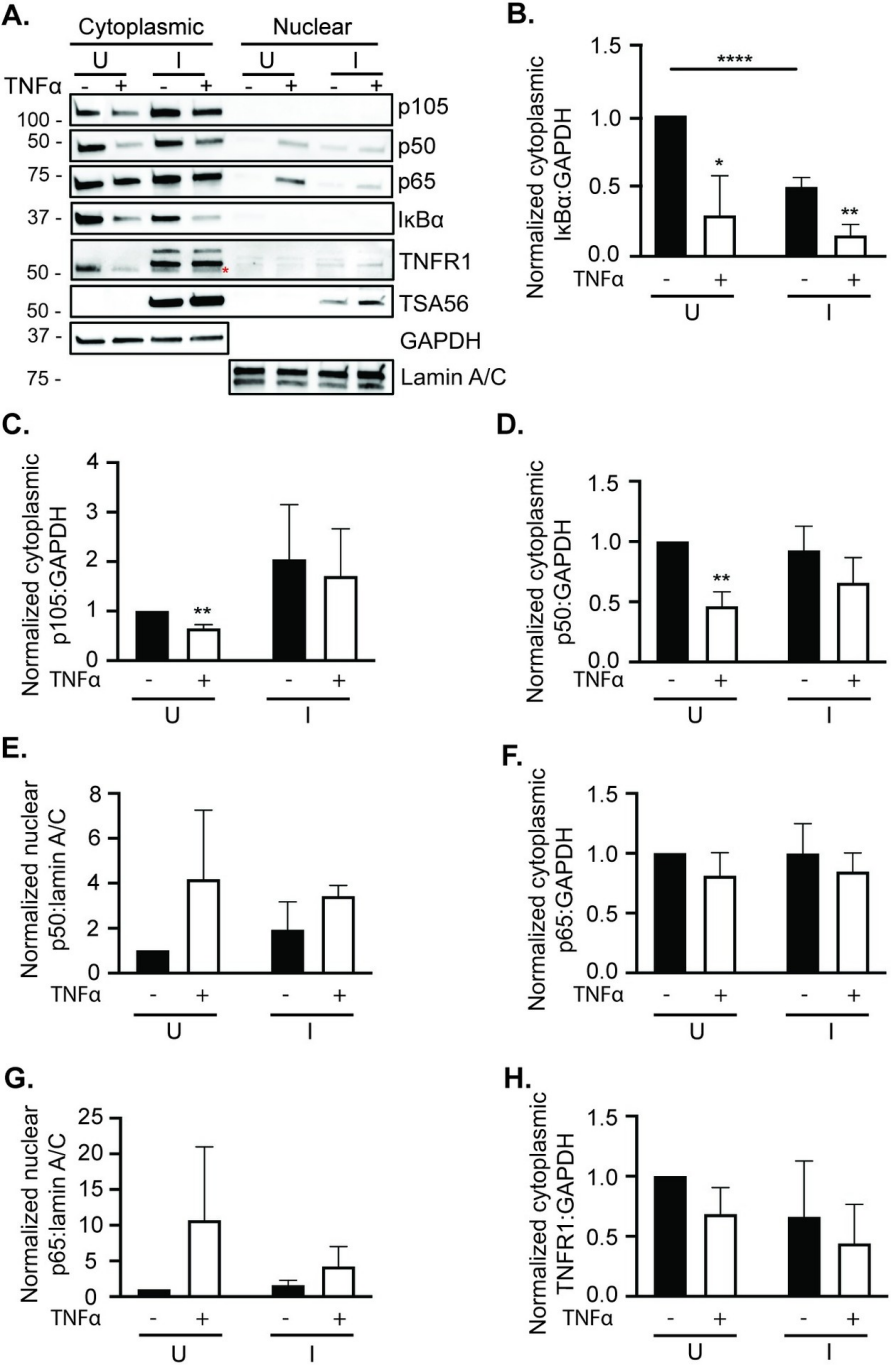
p105 levels remain elevated in *O*. *tsutsugamushi* infected cells treated with TNFα. HeLa cells were mock infected (U) or infected with *O*. *tsutsugamushi* at a MOI of 10 (I). At 72 h, the cells were treated with TNFα (+) or vehicle control (-). Cytoplasmic and nuclear fractions were analyzed by Western blot with the indicated antibodies (A). (B to G) Mean normalized ratios + SD of cytoplasmic IκBα:GAPDH (B), cytoplasmic p105:GAPDH (C), cytoplasmic p50:GAPDH (D), nuclear p50:lamin A/C (E), cytoplasmic p65:GAPDH (F), nuclear p65:lamin A/C (G), and TNFR1:GAPDH (H) from three independent experiments were calculated using densitometry. A red asterisk distinguishes the expected size for TNFR1 from the two bands of higher apparent molecular weights exclusively present in lysates of infected cells. Statistically significant (*, *P* < 0.05; **, *P* < 0.01; ****, *P* < 0.0001) values are indicated.

TNFα, which is detectable in sera from scrub typhus patients and animals experimentally infected with *O*. *tsutsugamushi*, signals via TNFα receptor 1 (TNFR1) to promote degradation of IκBα and p105 and thereby liberate NF-κB such that it can translocate into the nucleus to activate gene expression [12,13,38–49]. Given that *O*. *tsutsugamushi* inhibits NF-κB nuclear accumulation without impairing IκBα proteolysis [22], we rationalized that the pathogen might counter TNFα-induced p105 degradation. To test this hypothesis, HeLa cells were infected with *O*. *tsutsugamushi* at a MOI of 10 for 72 h followed by the addition of TNFα or vehicle control. Cytoplasmic and nuclear fractions were isolated and analyzed. GAPDH and lamin A/C were probed for as cytoplasmic and nuclear fraction loading controls, respectively). Both p105 and IκBα levels were reduced in uninfected control cells following TNFα exposure, indicating that conditions were apt for detecting degradation of both NF-κB inhibitory proteins (Fig 4A–4C). While p105 levels were reduced in both uninfected and infected cells upon TNFα treatment, its levels remained elevated in infected cells (Fig 4A and 4C). Cytoplasmic TNFR1 levels were not significantly altered in *O*. *tsutsugamushi* infected cells (Fig 4A and 4H), verifying that the increase in p105 observed for infected cells is not due to a defect in TNFR1 receptor expression or cellular levels. A reduction in TNFR1 levels in TNFα-stimulated uninfected cells was observed, which could be due the release of TNFR1 from the cell surface upon exposure to the cytokine [50]. In lysates of unstimulated and stimulated infected cells, TNFR1 antibody detected protein of the expected size for TNFR1 as well as two proteins of higher apparent molecular weights.

Next, we assessed if the *O*. *tsutsugamushi*-induced elevation in p105 levels correlates with retention of NF-κB p50:p65 in the cytoplasm. When NF-κB p50:p65 is absent from the nucleus, p50:p50 transcriptional inhibitory dimers remain in the nucleus bound to DNA [51]. Consistent with this phenomenon, p50 was found and p65 was barely detectable in nuclear fractions of *O*. *tsutsugamushi* infected cells whether or not TNFα was present (Fig 4A, 4E, and 4G). p65 was present in nuclear fractions of uninfected cells following the addition of TNFα, which, when combined with the p50 data, indicates that NF-κB p50:p65 translocated into the nucleus in the absence of infection, but was nearly abolished in infected cells (Fig 4A and 4D–4G). These data demonstrate that *O*. *tsutsugamushi* specifically stabilizes p105 to protect it from robust TNFα-induced degradation, enabling it to retain NF-κB p50:p65 in the cytoplasm.

### *O*. *tsutsugamushi* does not inhibit TNFα-stimulated phosphorylation of p105 Ser932 or alter IKKα or IKKβ levels

**Fig 5:**
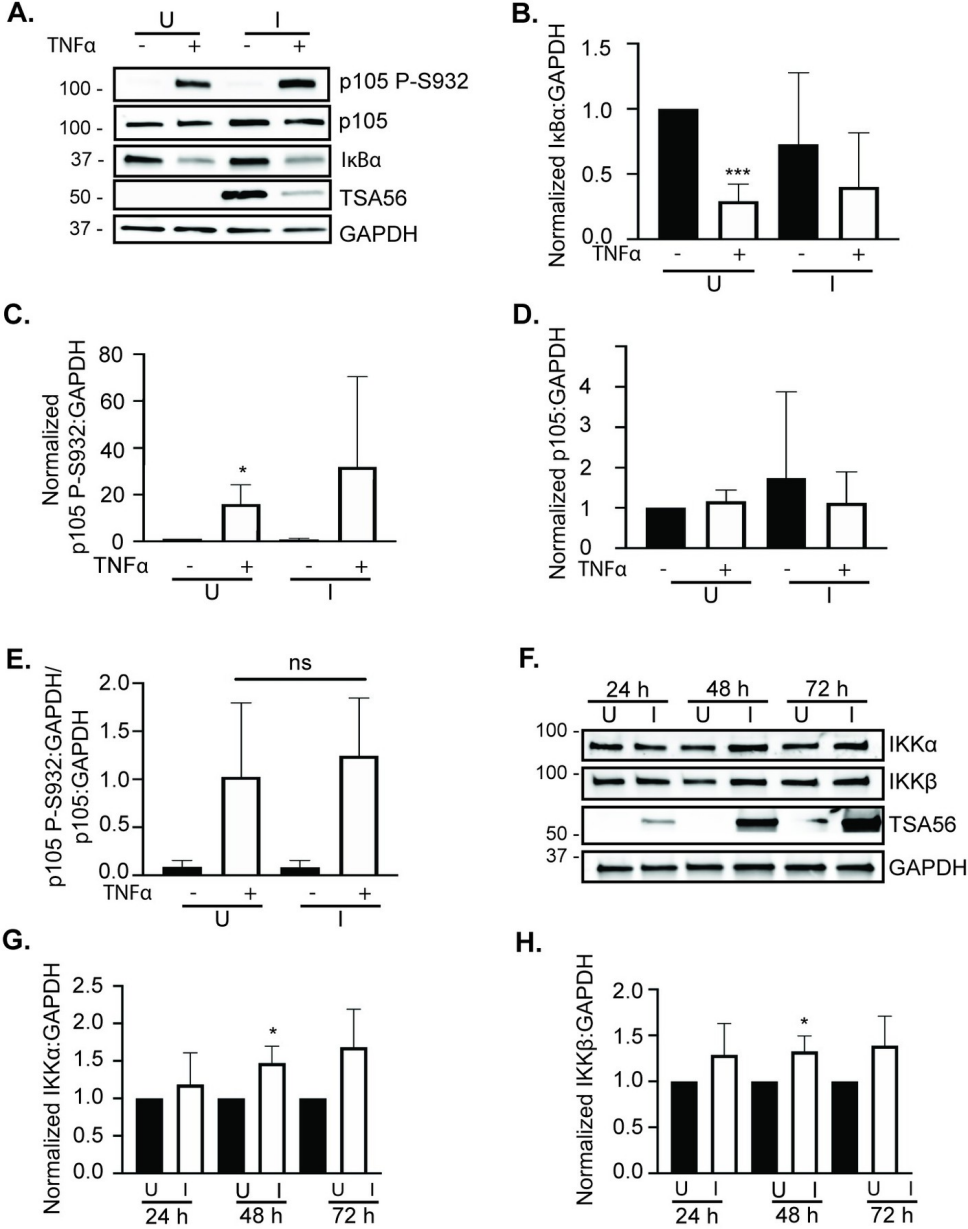
*O*. *tsutsugamushi* does not inhibit TNFα-stimulated phosphorylation of p105 Ser932 or reduce IKKα or IKKβ levels. (A to E) HeLa cells were mock infected (U) or infected with *O*. *tsutsugamushi* at a MOI of 10 (I). At 72 h, the cells were treated with calyculin A and TNFα (+) or vehicle control (-) for 5 min. (A) Whole cell lysates were collected and analyzed by Western blot using antibodies targeting p105, p105 phosphorylated Ser932 (P-S932), IκBα, TSA56, and GAPDH. Mean normalized ratios + SD of IκBα:GAPDH (B), p105 P-S932:GAPDH (C), p105:GAPDH (D), and P-S932:GAPDH/p105:GAPDH (E) from three separate experiments were calculated using densitometry. (F to H) Whole cell lysates of U and I HeLa cells recovered at 24, 48, and 72 h post-infection were subjected to Western blot analyses using the indicated antibodies (F). (G and H) Mean normalized ratios + SD of IKKα:GAPDH (G) or IKKβ:GAPDH (H) from three separate experiments in (F) were calculated using densitometry. Data are indicative of three independent experiments that yielded similar results. Statistically significant (*, *P* < 0.05; ***, *P* < 0.001) values are indicated.

TNFα activates the IKK complex, which includes IKKα and IKKβ [13], the latter of which phosphorylates IκBα and p105 to trigger their proteasomal degradation. Specifically, p105 Ser927 and Ser932 become phosphorylated [15,16]. Given the stability of p105 but not IκBα in TNFα treated *O*. *tsutsugamushi* infected cells, it was evaluated if the bacterium inhibits p105 serine phosphorylation. Infected and uninfected HeLa cells were exposed to TNFα at 72 h for 5 min. IκBα was rapidly degraded during this time frame, thus confirming that the treatment duration was sufficient to invoke classical NF-κB activation (Fig 5A and 5B). Screening with an antibody specific for phospho-Ser932 of p105 revealed that phosphorylation of this key serine was not altered in *O*. *tsutsugamushi* infected cells (Fig 5A and 5C–5E). Despite numerous attempts, we could not find an antibody that was capable of cleanly detecting p105 phospho-Ser927. Further analyses using antibodies against IKKα and IKKβ revealed that levels of both were not reduced during *O*. *tsutsugamushi* infection (Fig 5F–5H). Thus, the ability of *O*. *tsutsugamushi* to block TNFα-induced p105 proteolysis is not attributable to it inhibiting phosphorylation of p105 Ser932.

## Discussion

During the first few hours following *O*. *tsutsugamushi* invasion, the host cell responds by invoking NF-κB nuclear translocation [52,53]. This trend is reversed thereafter and for the duration of infection [22], indicating that the bacterium actively inhibits this pathway to retain NF-κB in the cytoplasm. The ability of *O*. *tsutsugamushi* to impair the NF-κB response is arguably key for its success not only as an endosymbiont in its natural arthropod vector and vertebrate reservoirs, but also as a human pathogen. The present study sheds light onto the responsible mechanism by demonstrating that *O*. *tsutsugamushi* promotes increased cellular levels of p105 to antagonize canonical NF-κB activation. The p105 increase in infected cells under steady state and TNFα-stimulated conditions is not due to an increase in *NFκB1* expression. Rather, it is caused, at least in part, by bacterial protein synthesis-dependent inhibition of p105 proteasomal degradation.

The molecular mechanism by which *O*. *tsutsugamushi* stabilizes p105 is unclear. The IKK complex, specifically IKKβ, phosphorylates p105 on Ser927 and Ser932 to create a high affinity binding site for β-transducin repeats-containing proteins (βTrCP), the receptor subunits for a Skp1-cullin1-F-box protein (SCF) E3-type ubiquitin ligase (SCF^βTrCP^) that ubiquitinates p105 on multiple lysine residues [17,54]. A similar phenotype to that observed for *O*. *tsutsugamushi* infected cells was reported in a study by Sriskantharajah and colleagues, in which they generated a mouse strain with IKK complex-targeted serine residues of p105 replaced by alanine [55]. Similar to that observed in *O*. *tsutsugamushi* infected cells, mutant p105 in these mice is resistant to IKK-induced degradation but can still be processed into p50. The high amount of p105 in T cells from these mice leads to cytoplasmic retention of NF-κB even under stimulation conditions [55]. Notably, just as detected in *O*. *tsutsugamushi* infected cells, p105 levels in T cells from these mice were elevated under resting conditions, while *NFκB1* transcript levels were unchanged relative to those of T cells from wild-type mice [55]. However, *O*. *tsutsugamushi* does not reduce IKKβ and IKKα levels or impair Ser932 phosphorylation. Moreover, IκBα, which is also serine-phosphorylated by IKKβ to facilitate SCF^βTrCP^-mediated ubiquitination [56,57], is effectively degraded upon TNFα stimulation of infected cells. Thus, *O*. *tsutsugamushi* does not target the IKK complex. Whether phosphorylation of other p105 serine residues, such as Ser927 or alternatively Ser903 and Ser907, the latter two of which at prime it for TNFα-induced proteolytic degradation [12], is altered in *O*. *tsutsugamushi* infected cells is unknown. The pathogen could also impede p105 degradation by interfering with SCF^βTrCP^ interaction or polyubiquitination of p105. It is worth noting that two anti-TNFR1 immunoreactive proteins of higher apparent molecular weights in addition to TNFR1 itself were exclusively present in infected cells under both unstimulated and TNFα stimulated conditions. These could merely be cross-reactive *O*. *tsutsugamushi* proteins. However, the alternative possibility that they are posttranslationally modified forms of TNFR1 that are induced by the bacterium should be considered and pursued as a line of future investigation.

While it cannot be absolutely ruled out that the increase in p105 levels in *O*. *tsutsugamushi* infected cells is not an indirect effect of the altered cellular physiology induced during infection, the tetracycline sensitivity of the phenomenon implies that one or more unidentified bacterial factors are responsible. The periodontal bacterial pathogen, *Porphyromonas gingivalis*, uses its SerB serine-protease to dephosphorylate NF-κB p65, but it does not act on p105 [58]. *O*. *tsutsugamushi* OTT_1962 (OtDUB) was recently structurally and functionally characterized as a deubiquitylase that, when expressed in recombinant form, exhibits a high affinity for and efficiently cleaves polyubiquitin chains [59]. As a role for the newly discovered OtDUB during infection has yet to be determined, it is plausible OtDUB deubiquitylates polyubiquitinated p105. Precedent for p105 stabilization was first reported for viruses. Poxviridae members encode orthologous ankyrin repeat-containing proteins (Anks) that bind to and stabilize p105 [60–63]. Cowpox virus in which the p105-targeting protein, CPX006, is replaced with an EGFP cassette stimulates p105 phosphorylation and degradation, NF-κB nuclear translocation, and proinflammatory cytokine production in THP-1 macrophages and NF-κB-dependent luciferase expression in a reporter cell line. It induces a massive inflammatory response and is significantly less lethal in mice versus wild-type cowpox and revertant virus [62]. Thus, poxviral Anks that stabilize p105 are sufficient to induce an anti-inflammatory state and disease progression. Some of these orthologs have been shown to also prevent IκBα degradation [62,64], which is in contrast to the specificity of *O*. *tsutsugamushi* for p105. Conspicuously, *O*. *tsutsugamushi* str. Ikeda encodes a large family of Anks [24,65]. When ectopically expressed, two of these effectors, Ank1 and Ank6, inhibit TNFα-induced NF-κB nuclear accumulation without blocking IκBα degradation. Ank1 and Ank6 inhibition of NF-κB involves their interaction with importin-α for translocation into the nucleus where they interfere with NF-κB-activation of gene expression and NF-κB binding to DNA, ultimately promoting exportin 1-dependent delivery of the transcription factor out of the nucleus [22]. Whether Ank1, Ank6, OtDUB or other *O*. *tsutsugamushi* effectors stabilize p105 and their mechanisms of action will be important to pursue in future investigations.

To the best of our knowledge, this study identifies *O*. *tsutsugamushi* as the first example of a bacterial pathogen that counters the canonical NF-κB pathway by stabilizing p105. When considered in the context that poxviruses also do so, an interesting example of convergent evolution emerges: p105 stabilization is a bottleneck in the NF-κB pathway that is effectively targeted by distinct intracellular microbes. Our findings uncover an additional layer in the multifaceted strategy by which *O*. *tsutsugamushi* subverts NF-κB in its attempt to counterbalance immune responses during scrub typhus. Given the importance of p105 as a suppressor of inflammation [12,21], this report also offers molecular insight that could potentially be exploited to therapeutically treat inflammatory disorders, autoimmunity, and cancers that are associated with uncontrolled NF-κB activation [13].
